# Adaptations to Concurrent Training in Combination with High Protein Availability: A Comparative Trial in Healthy, Recreationally Active Men

**DOI:** 10.1007/s40279-018-0999-9

**Published:** 2018-10-19

**Authors:** Baubak Shamim, Brooke L. Devlin, Ryan G. Timmins, Paul Tofari, Connor Lee Dow, Vernon G. Coffey, John A. Hawley, Donny M. Camera

**Affiliations:** 1 Exercise and Nutrition Research Program, Mary MacKillop Institute for Health Research, Australian Catholic University, Melbourne, VIC Australia; 2 School of Exercise Science, Australian Catholic University, Melbourne, VIC Australia; 3 Bond Institute of Health and Sport and Faculty of Health Sciences and Medicine, Bond University, Robina, QLD Australia

## Abstract

**Background:**

We implemented a high-protein diet (2 g·kg^−1^·d^−1^) throughout 12 weeks of concurrent exercise training to determine whether interferences to adaptation in muscle hypertrophy, strength and power could be attenuated compared to resistance training alone.

**Methods:**

Thirty-two recreationally active males (age: 25 ± 5 years, body mass index: 24 ± 3 kg·m^−2^; mean ± SD) performed 12 weeks of either isolated resistance (RES; *n *= 10) or endurance (END; *n *= 10) training (three sessions·w^−1^), or concurrent resistance and endurance (CET; *n *= 12) training (six sessions·w^−1^). Maximal strength (1RM), body composition and power were assessed pre- and post-intervention.

**Results:**

Leg press 1RM increased ~ 24 ± 13% and ~ 33 ± 16% in CET and RES from PRE-to-POST (*P *< 0.001), with no difference between groups. Total lean mass increased ~ 4% in both CET and RES from PRE-to-POST (*P *< 0.001). Ultrasound estimated *vastus lateralis* volume increased ~ 15% in CET and ~ 11% in RES from PRE-to-POST (*P *< 0.001), with no difference between groups. Wingate peak power relative to body mass displayed a trend (*P* = 0.053) to be greater in RES (12.5 ± 1.6 W·kg BM^−1^) than both CET (10.8 ± 1.7 W·kg BM^−1^) and END (10.9 ± 1.8 W·kg BM^−1^) at POST. Absolute VO_2peak_ increased 6.9% in CET and 12% in END from PRE-to-POST (*P *< 0.05), with no difference between groups.

**Conclusion:**

Despite high protein availability, select measures of anaerobic power-based adaptations, but not muscle strength or hypertrophy, appear susceptible to ‘interference effects’ with CET and should be closely monitored throughout training macro-cycles.

T*rials Registry*: This trial was registered with the Australian-New Zealand Clinical Trials Registry (ACTRN12617001229369).

**Electronic supplementary material:**

The online version of this article (10.1007/s40279-018-0999-9) contains supplementary material, which is available to authorized users.

## Key Points

**Table:** 

Little consideration has been given to the role of increased protein availability to facilitate anabolic adaptations to concurrent training.
Concurrent training combined with a high-protein diet does not impair gains in maximal strength, countermovement jump, squat jump, VO_2peak_, lean mass or muscle architectural changes compared to resistance or endurance training alone.
Despite optimal protein intake strategies, select measures of anaerobic power are compromised during a concurrent training block and should be monitored carefully.

## Introduction

The simultaneous development of strength, power and endurance adaptations is an attribute required by many athletes, particularly those involved in team sports [1]. Both muscular strength and cardiorespiratory fitness have been associated with lower declines in muscle function, chronic metabolic diseases and all-cause mortality [2, 3]. Incorporating both resistance- and endurance-based exercise into training programs, termed concurrent training, is therefore common practice in both athletic [4, 5] and clinical populations [6–8]. Further, World Health Organization global recommendations for physical activity for overall health and well-being in adults stipulate the performance of a combination of both resistance- and endurance-type exercises to improve cardiovascular and muscular fitness [9].

The principle of training specificity dictates that adaptations to chronic training are specific to the mode of exercise performed and result in distinct and divergent skeletal muscle phenotypes [10]. For example, endurance training improves skeletal muscle oxidative capacity and whole-body maximal oxygen uptake, leading to a more fatigue-resistant muscle [11, 12]. Conversely, strength training develops maximal force-generating capacity and skeletal muscle hypertrophy [13]. Given these vastly divergent adaptations, the simultaneous development of muscular endurance and strength/power with concurrent training presents a high degree of complexity in exercise prescription [14]. Indeed, findings from multiple studies demonstrate ‘interference’ in the magnitude of increase in hypertrophy, strength and power with concurrent training compared to resistance training undertaken in isolation [15–24], although these observations are not unequivocal [25–28].

Theoretical recommendations to prevent or reduce interference to strength adaptations have been formulated based on existing literature regarding concurrent training variables [29, 30], nutrition [31] and molecular biology [14, 32, 33]. It has been suggested that maximal strength and hypertrophy with concurrent training can be attained through implementing longer recovery periods (i.e. 6–24 h) between exercise sessions, minimizing endurance frequency to ≤ 3 days per week, integrating cycling rather than running as the endurance exercise mode (to minimize muscle damage) and incorporating post-exercise nutritional strategies [30]. With regard to nutrition, little consideration has been given to the role of increased protein availability to facilitate adaptations to concurrent training. We [34] have previously shown that protein ingestion following a single bout of concurrent exercise increased rates of muscle protein synthesis to similar levels observed when protein was ingested following resistance exercise [35]. Considering the importance for dietary protein to promote muscle growth and remodelling [36, 37] increased protein availability around concurrent training has the potential to reduce the interference effect of endurance exercise on skeletal muscle hypertrophy. Accordingly, we implemented a high-protein diet and other strategies to reduce the interference effect on maximal muscle strength, hypertrophy and power following 12 weeks of concurrent training compared to resistance training alone. We hypothesized that concurrent training under these conditions would result in no differences to the degree of adaptations made to (a) maximal strength, hypertrophy and power, compared to resistance training and (b) maximal aerobic capacity compared to endurance training.

## Methods

### Participants

Thirty-two young, healthy, recreationally active males (Table 1) who had not participated in a structured exercise program for ≥ 6 months preceding the study volunteered to participate. Participants were deemed healthy and eligible to participate based on their responses to a cardiovascular risk-factor questionnaire. The experimental procedures and risks associated with the study were explained to all participants prior to providing written informed consent. The study was approved by the Australian Catholic University Human Research Ethics Committee and was carried out in accordance with the standards set by the latest revision of the Declaration of Helsinki. This trial was registered with the Australian New Zealand Clinical Trials Registry (ACTRN12617001229369).

**Table 1:** Participant characteristics

	Training group					
	CET (*n* = 12)		RES (*n* = 10)		END (*n* = 10)	
	PRE	POST	PRE	POST	PRE	POST
Age (y)	26 ± 4	–	24 ± 6	–	24 ± 5	–
Height (cm)	177 ± 7	–	182 ± 8	–	179 ± 7	–
Mass (kg)	76.4 ± 10.2	79.3 ± 9.7^a^	75.5 ± 10.3	78.8 ± 11^a^	79.5 ± 9.3	81.5 ± 8.9^a^
BMI (kg·m^−2^)	24.4 ± 2.9	25.3 ± 2.6^a^	22.8 ± 2.8	23.8 ± 2.9^a^	24.8 ± 3.1	25.5 ± 2.9^a^

Values are presented as means ± SD

*BMI* body mass index, *CET* concurrent exercise training, *RES* resistance training, *END* endurance training

^a^*P* < 0.05 from PRE

### Experimental Design

**Fig. 1:**
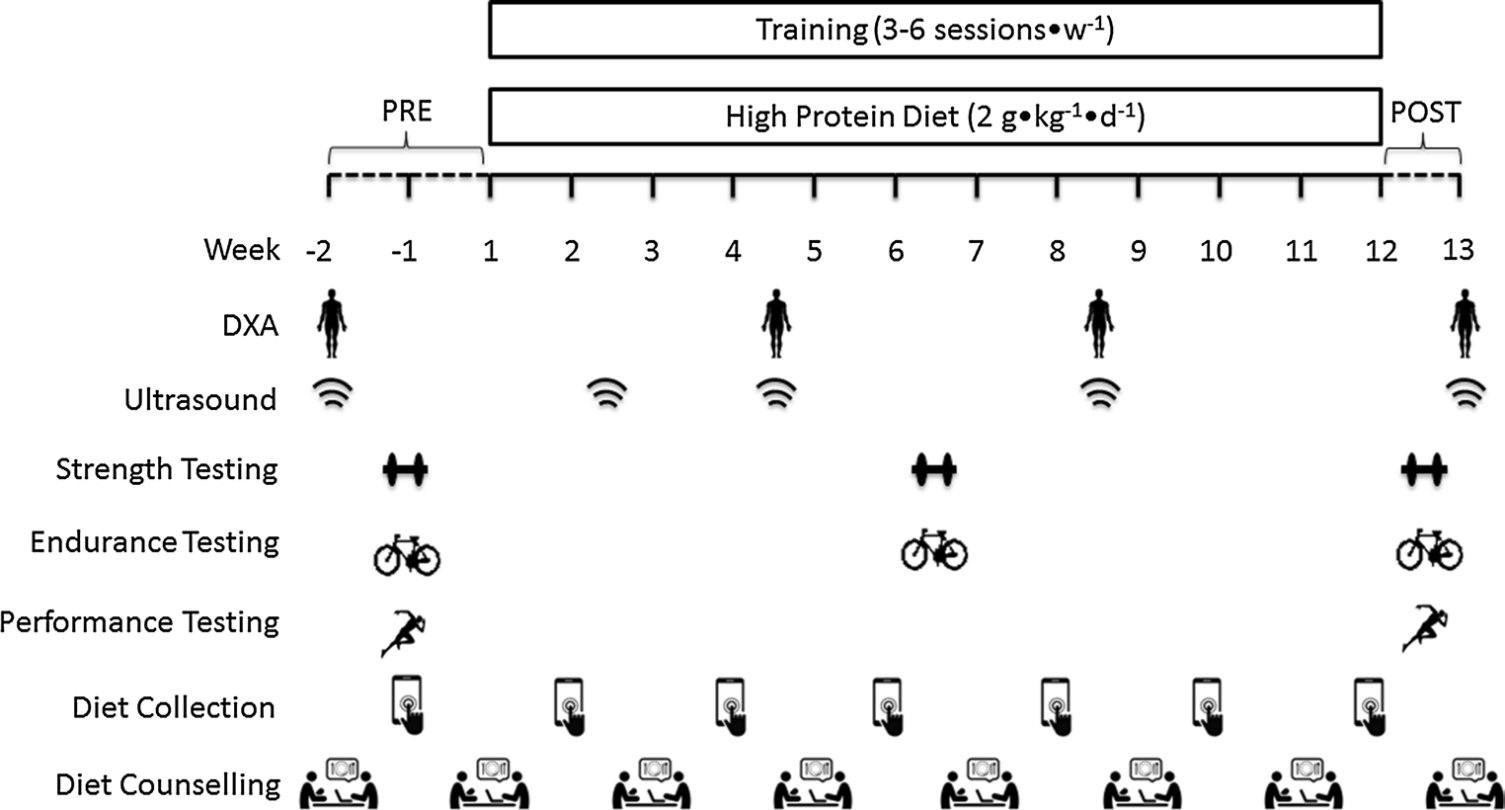
Schematic overview of study timeline

An overview of the study protocol is shown in Fig. 1. The study employed a parallel-groups design where participants were stratified according to lean body mass (LBM) and allocated to either a resistance only (RES; *n* = 10), endurance only (END; *n* = 10) or concurrent resistance and endurance exercise training (CET; *n* = 12) group for 12 weeks. For the duration of the intervention, all participants consumed a high-protein diet (2 g·kg^−1^·d^−1^). Participants first completed three preliminary testing days: on the first visit, body composition was assessed by whole-body dual-energy X-ray absorptiometry (DXA) and B-mode ultrasound to measure *vastus lateralis* (VL) architecture; on the second visit, participants performed tests for maximal aerobic capacity (VO_2peak_) and anaerobic power (Wingate) as well as familiarization of strength and jump performance measurements; on the third visit, participants completed an isometric mid-thigh pull, countermovement and squat jump, followed by 1-repetition maximum (1RM) testing. At this visit, participants also met with the study dietitian for an initial consultation to discuss food preferences as well as target protein and energy intakes prior to commencing the training intervention. Measurements of 1RM and VO_2peak_ were repeated at the end of week 6 to adjust training loads. At the end of week 12, participants were re-tested for VO_2peak_, Wingate, 1RM, isometric strength and power in the same order as baseline. All testing and training sessions were completed in the strength and performance lab under direct supervision of the same member of the research team.

### Exercise Training

For the duration of the intervention, participants in the RES and END group performed three non-consecutive days of training each week. Participants in the CET group trained 6 d·wk^−1^ and performed identical resistance and endurance programs on alternating days as those in the RES and END groups, respectively. This training pattern was implemented in the CET group based on current recommendations to perform resistance and endurance exercise on alternating days to maximize the potential for lower-body strength development [38, 39] and lengthen recovery time between sessions to minimize any potential interference between training modalities [30, 33]. All training programs were periodized to progressively modify the volume and intensity of training in order to provide an appropriate overload stimulus. Specific details of each training regime are described subsequently. Participants were encouraged to complete the designated training programs in their entirety with financial incentives provided for all three groups for largest pre- to post-intervention increases in 1RM (CET and RES) and VO_2peak_ (CET and END) [40].

### VO_2peak_ Testing

VO_2peak_ was determined during an incremental test to volitional fatigue on a Lode cycle ergometer (Excalibur sport, Lode, The Netherlands) [41]. Throughout the maximal test, participants breathed through a mouthpiece attached to a metabolic cart (TrueOne^®^ 2400, Parvomedics, USA) to determine O_2_ consumption. Maximum aerobic power (MAP) was determined as previously described [41] and was assessed prior to training, at the end of week 6, and upon completion of the 12-week training intervention. The MAP from pre-training and week 6 were used to prescribe loads for the endurance training.

### Strength Testing

Maximal strength was determined through 1RM for plate-loaded 45° incline leg press, bilateral knee extension, and bench press. Participants were demonstrated proper lifting technique prior to engaging in 1RM testing. Briefly, participants warmed up at a self-selected load for each movement until reaching a rating of perceived exertion (RPE) of ~ 8, using a Borg Category Ratio 10 scale [42], for a single repetition. Thereafter, a series of single repetitions were attempted, with 5 min recovery, until the maximal load possible for one repetition with full range of motion was determined. For the leg press, full range of motion was established as beginning with the knees in full extension (0°), performing 90° of knee flexion, and returning to full knee extension. For the knee extension, full range of motion was established as beginning with the knees in 90° of flexion and extending to full extension. For bench press, full range of motion was established as beginning with the arms in full elbow extension, lowering the barbell to the position of the chest until momentum has been terminated, and returning to full elbow extension. Participants were instructed to maintain contact of the head, shoulders, and buttocks with the bench and feet planted on the ground throughout the entire movement. The 1RM’s from pre-training and week 6 were used to prescribe training loads for the resistance-training program.

Maximal lower-body isometric strength (N and N·kg^−1^) was measured prior to, and upon completion of the 12-wk intervention using an isometric mid-thigh pull (IMTP) as previously described [43]. All data was collected on a force plate sampling at 600 Hz (400 Series Force Plate, Fitness Technologies, Australia) and analyzed using proprietary software (Ballistic Measurement System, Fitness Technology, Australia).

### Power Testing

Performance tests were conducted prior to and upon completion of the 12-week intervention to determine maximal anaerobic power output. Detailed descriptions of each measurement can be found in Online Resource 1.

### Body Composition

Total lean mass, as well as leg and upper-body lean mass, and fat mass were estimated by DXA (GE Lunar iDXA Pro, GE Healthcare; software: Encore 2009, version 16) pre-intervention, after weeks 4 and 8 of exercise training, and post-intervention following best practice guidelines [44].

## Architectural Assessment of Vastus Lateralis

Segmental muscle thickness, pennation angle, fascicle length and volume changes of the VL were assessed utilizing B-mode ultrasound at baseline, after weeks 2, 4, 8 and post-intervention (Online Resource 1).

### Resistance Training

Resistance training consisted of whole body exercises with a focus on the leg press, knee extension and bench press movements, with these exercises performed at an intensity of ~ 60–98% of 1RM. All exercises were separated by a 3-min between-set recovery period. If the participant was unable to achieve the prescribed number of repetitions, the weight was lowered by ~ 5–10% for the following set to uphold the repetition scheme. All sessions were preceded by a standardized warm up for the lower- or upper-body, irrespective of the training session. Progressive overload was applied by periodically manipulating the number of sets, repetitions, and relative intensity of load throughout the 12-week program. A detailed outline of the resistance-training program can be found in Online Resource 2.

### Endurance Training

Endurance cycle training was performed on Lode cycle ergometers and consisted of a mixture of a hill simulation ride of varying intensity (25–110% of MAP), moderate-intensity continuous training at 50% MAP, moderate-intensity interval training at 70% MAP and high-intensity interval training at 100% MAP. Moderate-intensity intervals were separated by a 60-s recovery period at ~ 40% MAP, to establish a 2.5:1 or 5:1 work-to-rest ratio. High-intensity intervals were separated by 20- to 60-s recovery periods, completed at ~ 40% MAP, to establish a 1:5, 1:2, or 1:1 work-to-rest ratio. All cycling sessions were preceded by 3–5 min of cycling at ≤ 50 W. Heart rate (HR), energy expenditure (EE) and RPE were collected at the end of each cycling stage. Progressive overload was applied by manipulating the number of intervals and relative intensity of load throughout the 12-week program. A detailed outline of the endurance-training program can be found in Online Resource 3.

### Diet

A free-living, high-protein (2 g·kg^−1^·d^−1^) eating plan was implemented over the 12-week intervention. Participants attended consultations with an Accredited Practicing Dietitian on a fortnightly basis and were provided with guidelines to reach protein and energy targets, including the distribution of protein intake throughout the day across 4–6 meals [45, 46] and the consumption of ~ 20–30 g of protein prior to sleep to maximize potential for muscle protein synthesis [47–49]. All participants were provided with ~ 34 g of whey protein (Pure Warrior 100% WPI, Swisse™, Australia) to be consumed upon cessation of every training session [50] and given a whey protein supplement (Whey Protein Concentrate, Bulk Nutrients, Australia) to consume as needed throughout the 12-week intervention.

Food records were kept daily by participants throughout the 12-wk intervention using mobile phone applications Easy Diet Diary (Xyris Software Pty Ltd, Australia, for participants with iPhones^®^, Apple Inc., USA; *n *= 20) and MyFitnessPal (MyFitnessPal Inc., USA, for participants with Android-based devices, Google Inc., USA; *n* = 12). All dietary intake data was analyzed using FoodWorks 8© (Xyris Software Pty Ltd, Australia) to ensure the same food database was used for all analysis. Diet records were analyzed for energy (kJ·kg^−1^), protein, carbohydrate, and fat (g·kg^−1^ for all macronutrients) to provide a daily average for the entire 12-week intervention. Complete dietary methods are detailed within the Online Resource 1.

### Statistical Analysis

An a priori power calculation (G*Power Version 3.1) using a *F* test, repeated measures, within-between interaction ANOVA revealed 30 participants were needed to detect a medium effect (Cohen’s *f* = 0.25) with a significance level of *α* = 0.05 and 80% power for change in lean body mass as measured by DXA [51]. Baseline characteristics and mean training variables (RPE, HR, time to complete set, training time, rest interval, and between session rest) were analyzed by one-way ANOVA (group). Strength, performance, VO_2peak_, body composition, training volume and diet data were analyzed by two-way ANOVA (group × time) with repeated measures. Where ANOVA revealed significance, *P* ≤ 0.05, a Student-Newman–Keuls post hoc test was conducted for pairwise multiple comparisons (SigmaPlot 12, Systat Software Inc., USA). When normality (Shapiro–Wilk) was violated, a nonparametric Kruskal–Wallis test was performed to determine differences between conditions where statistical significance differed from ANOVA (CMJ height and distal VL muscle thickness; SPSS v25, IBM, USA). All data are expressed as mean ± SD.

## Results

### Participant Characteristics

There were no differences between groups in baseline characteristics for height, BM, BMI or age (Table 1). There was a main effect for time for change in BM (*P* < 0.001). BM increased from PRE to POST by 3.9% in CET, 4.3% in RES and 2.7% in END (*P *< 0.001). There was a main effect for time for change in BMI (*P* < 0.001). BMI increased from PRE to POST by 3.9% in CET, 4.4% in RES and 2.7% in END (*P *< 0.001; Table 1).

### Body Composition

There was a main effect for time for change in total LBM (*P* < 0.001). Total LBM increased from PRE to POST by 3.8% in CET, 3.8% in RES and 2.9% in END (*P* < 0.001). There was a main effect for time for change in LLM (*P* < 0.001). LLM increased from PRE to POST by 5.4% in CET, 6% in RES, and 5.2% in END. There was a main effect for time for change in ULM (*P* < 0.001). ULM increased from PRE to POST by 2.9% in CET and 2.8% in RES (*P* < 0.01). Additionally, a main effect for time was observed for changes in fat mass (*P* = 0.009). Fat mass increased from PRE to POST by 9.5% in RES (*P* = 0.037; Table 2).

**Table 2:** Change in body composition throughout the 12-week training intervention measured by dual-energy X-ray absorptiometry (DXA)

Measure	Time			
	PRE	WK 4	WK 8	POST
Total lean mass (kg)				
CET	58.4 ± 6.34	59.8 ± 6.48^a^	60.6 ± 6.68^ab^	60.6 ± 6.46^ab^
RES	59.6 ± 6.71	60.9 ± 6.48^a^	61.6 ± 6.63^a^	61.9 ± 6.6^ab^
END	58.9 ± 5.45	60.0 ± 5.74^a^	60.0 ± 5.2^a^	60.6 ± 5.02^a^
Leg lean mass (kg)				
CET	20.7 ± 2.78	21.5 ± 2.79^a^	21.8 ± 2.78^a^	21.8 ± 2.72^a^
RES	20.6 ± 2.36	21.5 ± 2.29^a^	21.5 ± 2.34^a^	21.8 ± 2.17^a^
END	20.8 ± 2.28	21.6 ± 2.47^a^	21.5 ± 2.27^a^	21.9 ± 2.38^ac^
Upper lean mass (kg)				
CET	34.8 ± 3.68	34.9 ± 3.76^a^	35.4 ± 3.97^a^	35.3 ± 3.82^a^
RES	35.5 ± 4.34	35.9 ± 4.27	36.5 ± 4.39^a^	36.5 ± 4.48^a^
END	34.6 ± 3.37	34.9 ± 3.58	35.1 ± 3.24	35.2 ± 2.97
Fat mass (kg)				
CET	15.4 ± 6.67	15.3 ± 6.61	15.7 ± 6.37	16.2 ± 5.76
RES	13.2 ± 5.84	13.6 ± 5.79	13.8 ± 5.95	14.3 ± 6.17^a^
END	17.9 ± 6.36	18.2 ± 6.24	18.2 ± 6.19	18.4 ± 6.06

Values are presented as means ± SD

*CET* concurrent exercise training, *RES* resistance training, *END* endurance training

^a^*P* < 0.05 from PRE

^b^*P* < 0.05 from WK4

^c^*P* < 0.05 from WK8

### Vastus Lateralis Architecture

There was a main effect for time (*P* < 0.001) and a trend for a group by time interaction (*P* = 0.051) for change in proximal VL muscle thickness. Proximal VL muscle thickness increased from PRE to POST by 14.9% in CET, 15.7% in RES, and 5.8% with END (*P* < 0.01). Proximal VL thickness at POST was greater in CET and RES compared to END (*P* < 0.05). There was an interaction for group by time for change in midpoint VL muscle thickness (*P* < 0.001). Midpoint VL muscle thickness increased from PRE to POST by 17.5% in CET, 13.7% in RES, and 9.8% in END (*P* < 0.001). Midpoint VL thickness at POST was greater in CET and RES compared to END (*P* < 0.05). Distal VL muscle thickness did not change (*P* = 0.054; Online Resource 4).

There was a main effect for time for change in proximal VL pennation angle (*P* < 0.001). Proximal VL pennation angle increased from PRE to POST by 17.2% in CET, 15.8% in RES, and 15.4% in END (*P* < 0.001). There was a main effect for time for change in midpoint VL pennation angle (*P* < 0.001). Midpoint VL pennation angle increased from PRE to POST by 12.4% in CET, 12.2% in RES, and 13.9% in END (*P* < 0.001). There was a main effect for time for change in distal VL pennation angle (*P* < 0.001). Distal VL pennation angle increased from PRE to POST by 12.3% in CET, 19% in RES, and 13.5% in END (*P* ≤ 0.005; Online Resource 4). There was a main effect for group for change in proximal VL fascicle length (*P* < 0.001). Proximal VL fascicle length decreased from PRE to POST by 6.3% in END (*P* = 0.024). Proximal VL fascicle length was significantly greater at POST in CET (9.3 ± 0.8 cm) compared to END (8.5 ± 0.6 cm; *P* = 0.036). There was a main effect for group for change in midpoint VL fascicle length (*P* = 0.004). Midpoint VL fascicle length was greater at PRE in CET (9 ± 0.9 cm) and RES (8.9 ± 0.8 cm) compared to END (8.3 ± 0.9 cm; *P* < 0.05). Midpoint VL fascicle length was also greater at POST in CET (9.5 ± 0.5 cm) and RES (9.1 ± 0.5 cm) compared to END (8.1 ± 0.6 cm; *P* < 0.01). There was a main effect for time for change in distal VL fascicle length (*P* = 0.031). Distal VL fascicle length increased from PRE to POST by 10.4% in CET (*P* = 0.024; Online Resource 4).

There was a main effect for time (*P* < 0.001) and a trend (*P* = 0.051) for a group by time interaction for changes in approximated VL muscle volume. Estimated VL muscle volume increased from PRE to POST by 15.3% in CET, 11.4% in RES, and 7.8% in END (*P* < 0.001; Online Resource 4).

### Strength

**Fig. 2:**
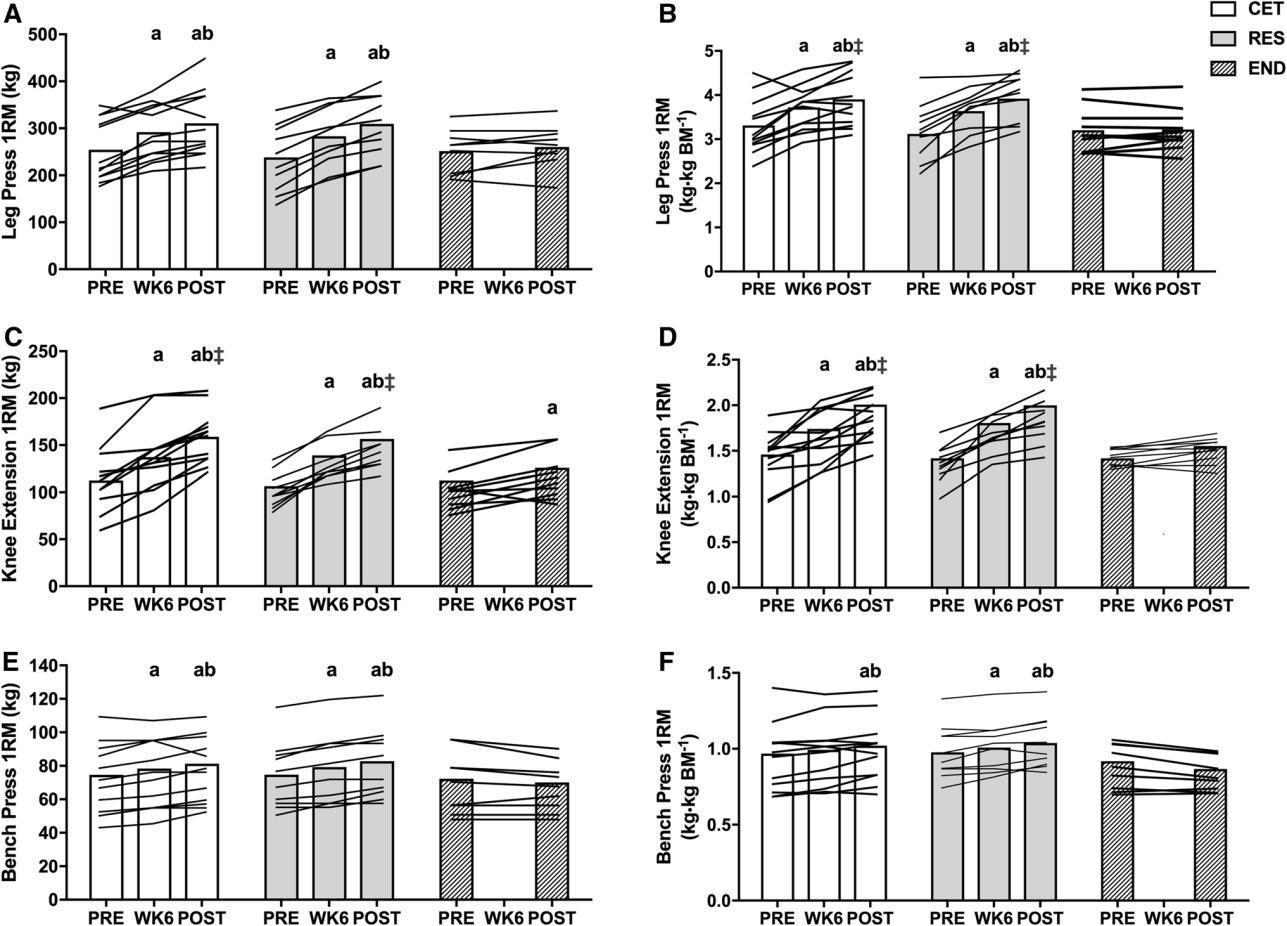
Changes to 1-repetition maximum (1RM) strength throughout the 12-week intervention for absolute (**a**–**c**) and relative (**d**–**f**) leg press, knee extension and bench press (END, *n* = 9). Values are presented as individual data with group mean. a = *P* < 0.05 from PRE. b = *P* < 0.05 from WK6. ǂ = *P* < 0.05 from END at time point. *CET* concurrent exercise training, *RES* resistance training, *END* endurance training

There was an interaction for group by time for change in absolute (*P* < 0.001) and relative to BM (*P* < 0.001) leg press 1RM. Absolute leg press 1RM increased in CET by 16.4% from PRE to WK6, 6.4% from WK6 to POST, and 23.7% from PRE to POST (*P* < 0.01). For RES, leg press 1RM increased 21.2% from PRE to WK6, 9.9% from WK6 to POST, and 33.4% from PRE to POST (*P* ≤ 0.001; Fig. 2a). Relative leg press 1RM was greater at POST in both CET (3.9 ± 0.6 kg·kg BM^−1^) and RES (3.9 ± 0.5 kg·kg BM^−1^) compared to END (3.2 ± 0.6 kg·kg BM^−1^; *P* = 0.05; Fig. 2b).

There was an interaction for group by time for change in absolute (*P* < 0.001) and relative to BM (*P* < 0.001) knee extension 1RM. Absolute knee extension 1RM increased in CET by 24.7% from PRE to WK6, 18.7% from WK6 to POST, and 48.7% from PRE to POST (*P* < 0.001). For RES, knee extension 1RM increased 32.2% from PRE to WK6, 12.7% from WK6 to POST, and 49.4% from PRE to POST (*P* ≤ 0.001). Knee extension 1RM was also greater at POST in both CET (159 ± 29 kg) and RES (157 ± 25 kg) compared to END (126 ± 21 kg; *P* < 0.05). Knee extension 1RM increased 12.5% from PRE to POST in END (*P* = 0.024; Fig. 2c); however, relative to BM, knee extension 1RM remained unchanged from PRE to POST in END (*P* = 0.122; Fig. 2d).

There was an interaction for group by time for change in absolute (*P* < 0.001) and relative to BM (*P* < 0.001) bench press 1RM. Absolute bench press 1RM increased in CET by 5.6% from PRE to WK6, 4.6% from WK6 to POST, and 10.4% from PRE to POST (*P* < 0.05). For RES, bench press 1RM increased 6% from PRE to WK6, 4.9% from WK6 to POST, and 11.3% from PRE to POST (*P* < 0.01; Fig. 2e). Relative bench press 1RM for CET trended towards an increase at WK6 (*P *= 0.055), and increased from both PRE and WK6 by POST (*P* < 0.05; Fig. 2f).

There was an interaction for group by time for change in IMTP peak force (*P* = 0.045). IMTP peak force increased from PRE to POST by 10.1% in CET and 9.6% in RES (*P* < 0.01; Online Resource 5). There was main effect for time for change in IMTP peak force relative to BM (*P* = 0.045). Relative IMTP increased from PRE to POST by 6.8% in CET and 6% in RES (*P* < 0.05; Online Resource 5).

### Power Testing

**Fig. 3:**
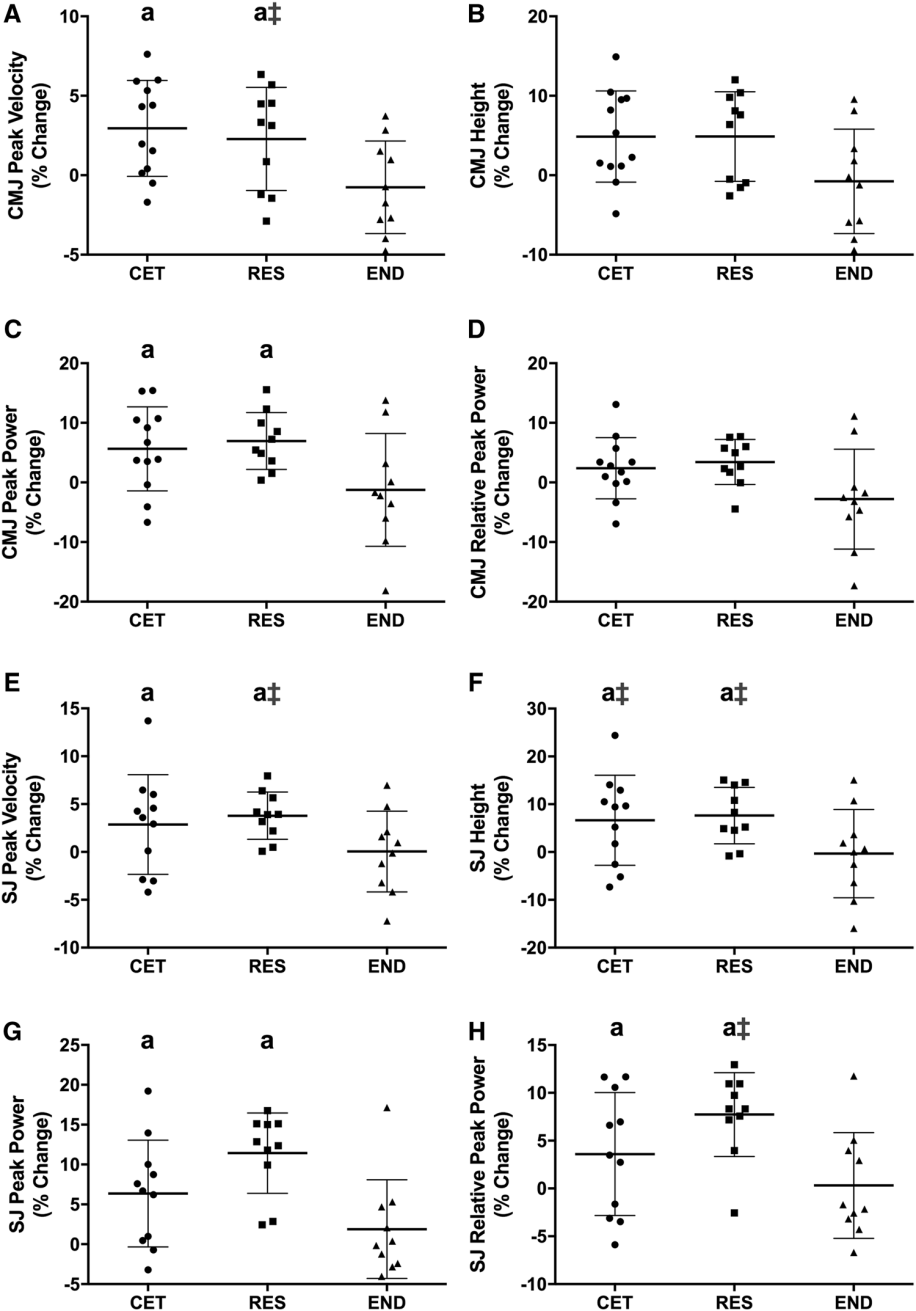
Change to countermovement jump (CMJ) and squat jump (SJ). Percent changes from PRE to POST for CMJ are presented for (**a**) peak velocity, (**b**) height, (**c**) peak power, and (**d**) relative peak power. Percent changes from PRE to POST for SJ are presented for (**e**) peak velocity, (**f**) height, (**g**) peak power, and (**h**) relative peak power. Values are presented as individual data with group mean ± SD. a = *P* < 0.05 from PRE. ǂ = *P* < 0.05 from END at POST. *CET* concurrent exercise training, *RES* resistance training, *END* endurance training

There was an interaction for group by time for change in CMJ peak velocity (*P* = 0.021). CMJ peak velocity increased from PRE to POST by 3% in CET and 2.3% in RES (*P *< 0.05; Fig. 3a). CMJ peak velocity at POST was greater in RES (2.95 ± 0.17 m·s^−1^) compared to END (2.68 ± 0.27 m·s^−1^; *P* = 0.027). CMJ height did not change (*P* = 0.089; Fig. 3b). There was an interaction for group by time for change in CMJ peak power (*P* = 0.047). CMJ peak power increased from PRE to POST by 5.6% in CET and 7% in RES (*P* < 0.05; Fig. 3c). There was an interaction for group by time for change in CMJ peak power relative to BM (*P* = 0.047); however, post hoc analysis revealed no changes to CMJ relative peak power across all groups (Fig. 3d).

There was a main effect for time for changes in SJ peak velocity (*P* = 0.006). SJ peak velocity increased from PRE to POST by 2.9% in CET and 3.8% in RES (*P *< 0.05; Fig. 3e). SJ peak velocity at POST was greater in RES (2.78 ± 0.23 m·s^−1^) compared to END (2.51 ± 0.23 m·s^−1^; *P* = 0.037). There was an interaction for group by time for change in SJ height (*P* = 0.047). SJ height increased from PRE to POST by 6.6% in CET and 7.6% in RES (*P *< 0.05; Fig. 3f). SJ height at POST was greater in CET (37.3 ± 4.86 cm) and RES (39.6 ± 6.77 cm) compared to END (32.2 ± 6.09 cm; *P *< 0.05). There was an interaction for group by time for change in SJ peak power (*P* = 0.005). SJ peak power increased from PRE to POST by 6.4% in CET and 11.4% in RES (*P* < 0.01; Fig. 3g). There was an interaction for group by time for change in SJ peak power relative to BM (*P* = 0.012). SJ relative peak power increased from PRE to POST by 3.6% in CET and 7.7% in RES (*P* < 0.05; Fig. 3h). SJ relative peak power was greater at POST in RES (53.4 ± 7.16 W·kg BM^−1^) compared to END (45.7 ± 6.9 W·kg BM^−1^; *P* = 0.047).

### Vo_2peak_

**Fig. 4:**
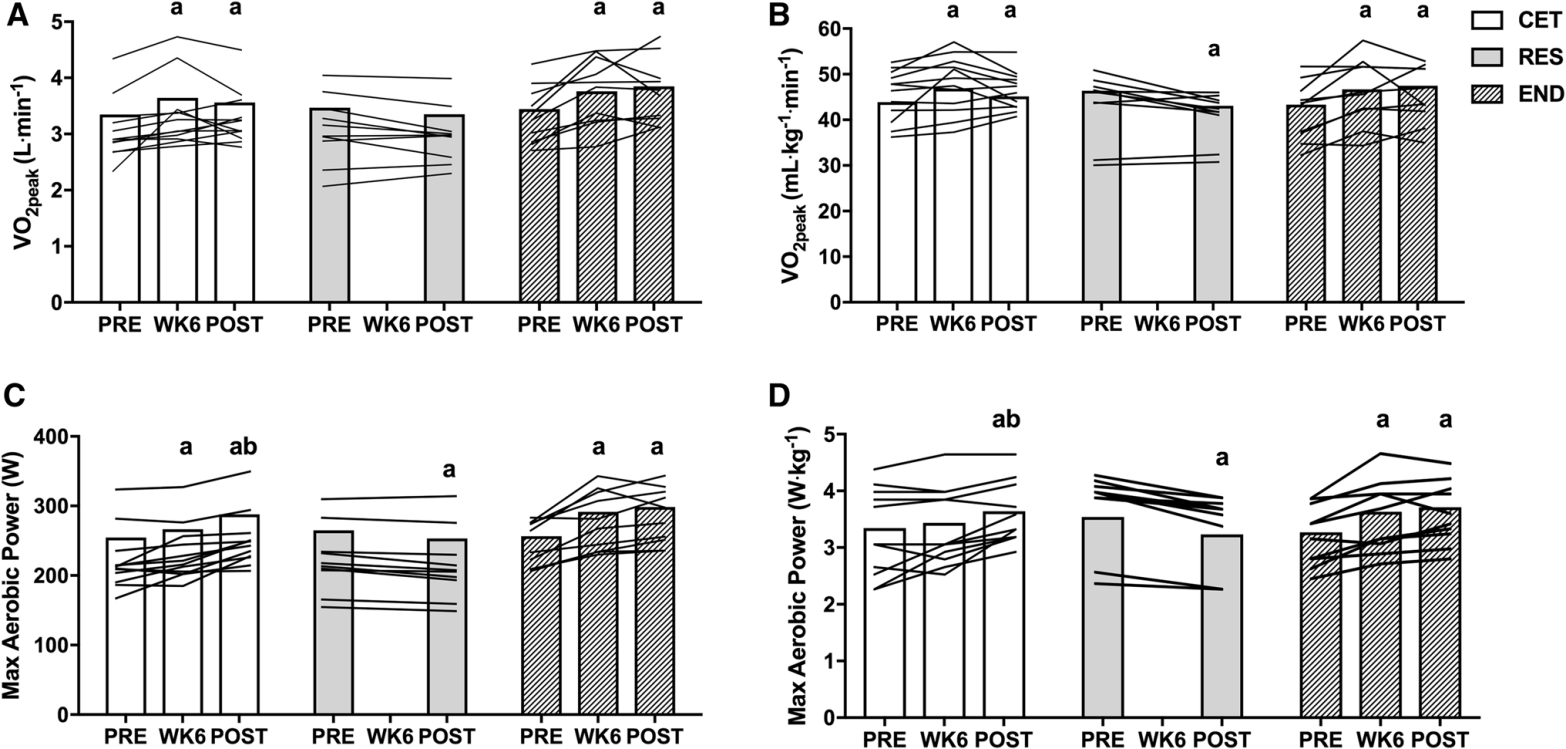
Changes to (**a**) absolute and (**b**) relative VO_2peak_ as well as (**c**) absolute and (**d**) relative maximum aerobic power throughout the 12-week intervention. Values are presented as individual data with group mean. a = *P* < 0.05 from PRE. b = *P* < 0.05 from WK6. *CET* concurrent exercise training, *RES* resistance training, *END* endurance training

There was an interaction for group by time for change in absolute (*P* < 0.001) and relative to BM (*P* < 0.001) VO_2peak_. Absolute VO_2peak_ (L·min^−1^) increased in CET by 9.1% from PRE to WK6, and 6.9% from PRE to POST (*P* < 0.05). For END, absolute VO_2peak_ increased 10.6% from PRE to WK6, and 12% from PRE to POST (*P* < 0.001); however, there was no difference between CET and END at POST (*P* = 0.208; Fig. 4a). Relative VO_2peak_ in CET increased by 6.9% from PRE to WK6 (*P* = 0.029), but did not change from PRE to POST (*P* = 0.272). For RES, relative VO_2peak_ decreased 4.8% from PRE to POST (*P* = 0.016). In contrast, relative VO_2peak_ increased in END by 9.1% from PRE to WK6, and 9.8% from PRE to POST (*P* < 0.005); however, there was no difference between CET and END at POST (*P* = 0.415; Fig. 4b).

There was an interaction for group by time for change in absolute (*P* < 0.001) and relative to BM (*P* < 0.001) MAP. Absolute MAP increased in CET by 5.3% from PRE to WK6, 8.5% from WK6 to POST, and 14% from PRE to POST (*P* < 0.05). For RES, absolute MAP decreased by 4.5% from PRE to POST (*P* = 0.015). For END, absolute MAP increased 13.6% from PRE to WK6, and 16.4% from PRE to POST (*P* < 0.001); however, there was no difference between CET and END at POST (*P* = 0.605; Fig. 4c). Relative MAP increased in CET by 7.1% from WK6 to POST (*P* = 0.002), and 9.8% from PRE to POST (*P* < 0.001). For RES, relative MAP decreased by 8.4% from PRE to POST (*P* < 0.001). For END, relative MAP increased by 11.2% from PRE to WK6 (*P* < 0.001), and 13.5% from PRE to POST (*P* < 0.001); however, there was no difference between CET and END at POST (*P* = 0.830; Fig. 4d).

### Wingate Indices

**Fig. 5:**
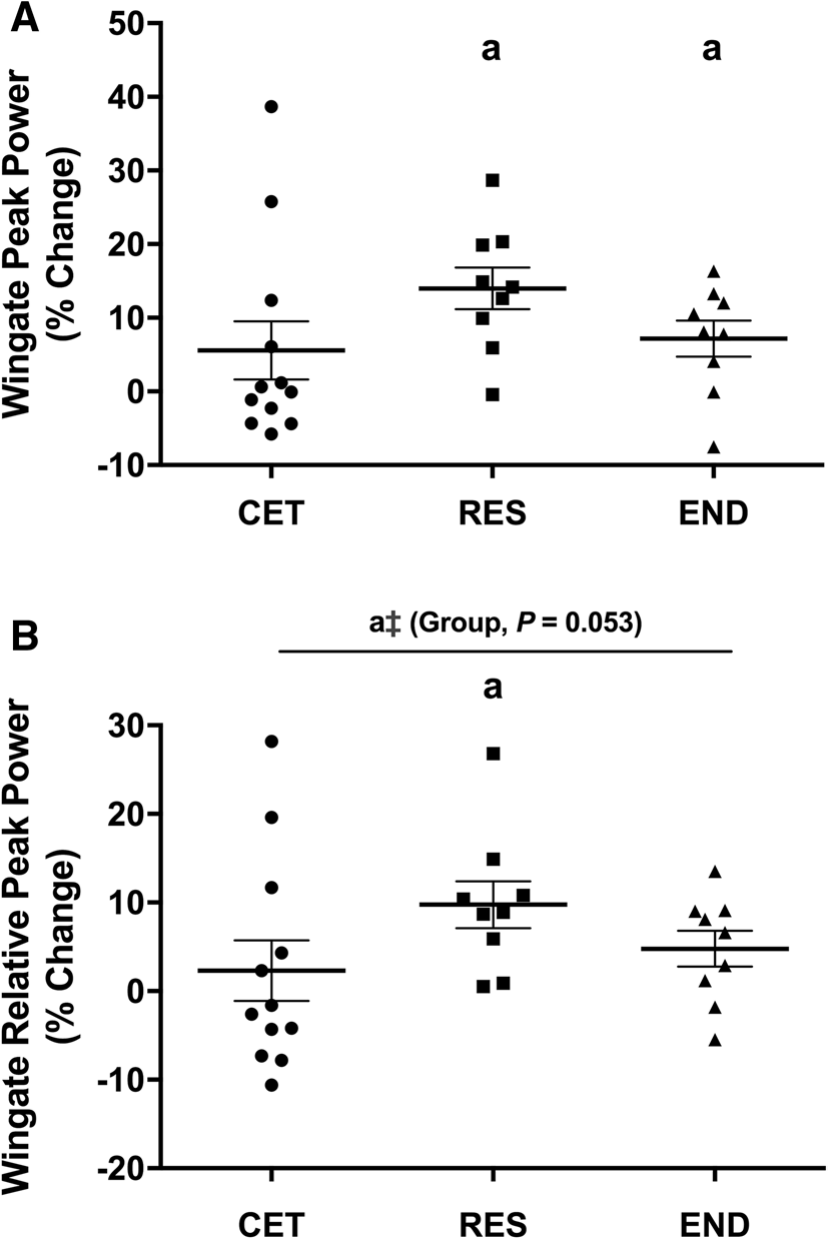
Change to (**a**) absolute and (**b**) relative Wingate peak power. Values are presented as percent change from PRE to POST and presented as individual data with group mean ± SD (RES, *n* = 9; END, *n* = 9). a = *P* < 0.05 from PRE. ǂ = *P* < 0.05 from END at POST. * = *P* < 0.05 from CET at POST. *CET* concurrent exercise training, *RES* resistance training, *END* endurance training

There was main effect for time (*P* < 0.001), but not group (*P* = 0.487) for training-induced change in Wingate peak power. Wingate peak power increased from PRE to POST by 14% in RES and 7.2% in END (*P* < 0.05) while there was no change in CET (*P* = 0.115; Fig. 5a). A main effect for time (*P* = 0.001) and a trend for group (*P* = 0.053) was observed for change in Wingate peak power when expressed relative to BM. Wingate relative peak power increased from PRE to POST by 9.8% in RES (*P* = 0.002). Wingate relative peak power at POST was greater in RES (12.5 ± 1.6 W·kg BM^−1^; *P* < 0.05) compared to both CET (10.8 ± 0.8 W·kg BM^−1^) and END (10.9 ± 1.8 W·kg BM^−1^; Fig. 5b).

### Training Volume and Variables

There was main effect for time for change in resistance training volume (*P* < 0.001); however, post hoc analysis revealed no difference between CET and RES across the training intervention (*P* = 0.385; Online Resource 6). Similarly, one-way ANOVA revealed no differences between CET and RES for average time to complete set (*P* = 0.564), between-set rest interval (*P* = 0.915), or RPE (*P* = 0.838; Online Resource 7). There was main effect for time for change in endurance training volume (*P* < 0.001); however, post hoc analysis revealed no difference between CET and END across the training intervention (*P* = 0.708; Online Resource 6). One-way ANOVA revealed no difference in average training hours (*P* = 0.488) or HR (*P* = 0.222) between CET and END across the training intervention. However, average RPE was 10% higher in CET compared to END (*P* < 0.001). Recovery time between sessions (Online Resource 7) was significantly less in CET (23.6 h; *P* < 0.001) compared to both RES (47.7 h) and END (48 h).

### Diet

There was main effect for time (*P* = 0.005) and group (*P* = 0.026) for change in energy intake. Energy intake was significantly greater at baseline in RES (~ 11,300 kJ) compared to END (~ 8780 kJ; *P* = 0.007). Average daily energy intake during training increased from baseline by 12.5% in CET and 20.1% in END (*P* < 0.05). There was no difference in energy intake across conditions during the training intervention (CET = ~ 11,400 kJ; RES = ~ 11,700 kJ; END = ~ 10,600 kJ; *P* = 0.348). There was main effect for time (*P* < 0.001) and group (*P* = 0.046) for change in protein intake. Protein intake was significantly greater at baseline in CET (1.6 g·kg^−1^·d^−1^) and RES (1.7 g·kg^−1^·d^−1^) compared to END (1.3 g·kg^−1^·d^−1^: *P* < 0.05). Average daily protein intake during training increased from baseline by 40.6% in CET, 26.3% in RES, and 61.7% in END (*P* < 0.005). Carbohydrate intake was greater at baseline in RES (4.1 g·kg^−1^·d^−1^; *P* = 0.044) than END (3.1 g·kg^−1^·d^−1^); however, no effect for group (*P* = 0.072), time (*P* = 0.6), or group by time (*P* = 0.116) was observed. There was a main effect for group for fat intake (*P* = 0.004). Fat intake was significantly greater at baseline in RES (1.5 g·kg^−1^·d^−1^; *P* < 0.05) compared to both CET (1.1 g·kg^−1^·d^−1^) and END (1 g·kg^−1^·d^−1^; Table 3).

**Table 3:** Average dietary intake at baseline and throughout the 12-week training intervention

	Time	
	Baseline	Training
Energy (kJ·d^−1^)		
CET	10,200 ± 2360	11,400 ± 1490^a^
RES	11,300 ± 1780^ǂ^	11,700 ± 1360
END	8780 ± 1900	10,600 ± 1630^a^
Protein (g·kg^−1^·d^−1^)		
CET	1.6 ± 0.51^ǂ^	2.2 ± 0.17^a^
RES	1.7 ± 0.47^ǂ^	2.1 ± 0.17^a^
END	1.3 ± 0.48	2.0 ± 0.13^a^
Carbohydrate (g·kg^−1^·d^−1^)		
CET	3.5 ± 0.81	3.8 ± 1.00
RES	4.1 ± 1.04^ǂ^	3.6 ± 0.65
END	3.1 ± 0.88	3.0 ± 0.63
Fat (g·kg^−1^·d^−1^)		
CET	1.1 ± 0.28	1.2 ± 0.34
RES	1.5 ± 0.4*^ǂ^	1.5 ± 0.25
END	1.0 ± 0.32	1.2 ± 0.3

Values are presented as means ± SD

*CET* concurrent exercise training, *RES* resistance training, *END* endurance training

^a^*P* < 0.05 from Baseline

^ǂ^*P* < 0.05 from END at time point

*P < 0.05 from CET at time point

## Discussion

This is the first investigation to compare the effects of long-term (i.e. 12 weeks) concurrent training in combination with a high-protein diet on a broad range of adaptations in skeletal muscle. We show that concurrent resistance and endurance training when performed 3 d·wk^−1^ on alternate days, in combination with a high protein availability, does not impair gains in maximal strength, lean mass or aerobic capacity compared to resistance training alone. In contrast, concurrent training may attenuate specific lower-body developments to maximal anaerobic power output compared to resistance training alone and should be closely monitored. Our findings provide novel information for practitioners for prescribing evidence-based recommendations for concurrent training strategies capable of maximizing strength, hypertrophy and aerobic adaptation responses.

The concurrent training ‘interference effect’ in strength and power adaptations was first observed by Hickson [15]. Since that seminal study, numerous investigations [18–23] have confirmed observations of compromised strength gains when strength and endurance training are undertaken concurrently. In contrast, others [25, 52–57] have reported little or no impairments to strength when undertaking concurrent training. Such disparities may be attributed a number of factors including volume, intensity and frequency of sessions, as well as training status of participants, modes of exercise being employed, and duration of intervention [14]. Indeed, the duration of many studies is less than the 8-week time point at which the interference effect was first observed [25, 53]. It has been proposed that maximal muscle growth with concurrent training can be achieved by implementing appropriate recovery periods (i.e. 6–24 h) between exercise sessions, incorporating post-exercise nutritional strategies, minimizing endurance/aerobic exercise to 2 × 3 d·wk^−1^, and integrating cycling compared to running as the endurance exercise mode [30, 31, 33]. To address some of these issues and determine whether they might reduce the interference effect, we undertook a comprehensive study protocol in which, for the first time in a concurrent training paradigm, we incorporated the aforementioned recommendations along with a ‘high’ protein diet over a 12-week training intervention to determine whether interferences in muscle strength, power and hypertrophy could be offset by following these guidelines.

The first major finding of our work was that muscle strength and hypertrophy with CET were not compromised compared to RES alone. We propose that the lack of ‘interference’ effect was due to several interrelated factors implemented in our training intervention. Firstly, the CET group performed resistance and endurance sessions on alternate days to allow a minimum of ~ 24 h recovery between bouts. Both proximity [16, 58, 59] and order [60–62] of endurance and resistance exercises performed during a concurrent training program can compromise muscle activation and force development, which can hinder the intensity and effort at which subsequent resistance exercise is performed, leading to reduced dynamic strength gains [38]. Increasing the recovery time or performing individual modes of exercise on separate days altogether [27, 58, 59, 63–65] alleviates residual fatigue and prevents impairments to force development. Our findings provide supporting evidence of the importance within a concurrent program of performing divergent modes of exercise on alternate days to promote strength adaptations.

The volume of endurance exercise performed can also impact strength adaptations [66]. Findings from a meta-analysis of 21 studies revealed a positive association between duration (length of session) and frequency (days per week) of endurance exercise and the degree of interference to strength gains [29]. However, concurrent training incorporating work-matched moderate-intensity continuous (MICT) or high-intensity interval training (HIT) attenuates lower-body strength by a similar magnitude [23], indicating that training intensity may not mediate interferences to maximal strength. In the current study, endurance training consisted of a combination of MICT and HIT cycling, with sessions lasting, on average, ~ 30 min for 3 d·wk^−1^. This combination, which significantly increased VO_2peak_, effectively circumvented any interference to strength development over 12 weeks of concurrent training. In this regard, the increase in absolute VO_2peak_ observed with CET is in line with previous literature [15, 21–23, 61, 67–70]. However, relative VO_2peak_ was only increased from baseline at WK6 in CET, while END demonstrated improvements from baseline at both WK6 and POST. As both CET and END performed the same volume of cycling, and increased BM similarly throughout the intervention, it is unclear why an increase in relative VO_2peak_ was not observed at POST in CET. Notably, CET displayed a higher average RPE during training, perhaps indicating a greater degree of residual fatigue. Nonetheless, both absolute and relative MAP increased from WK6 to POST in CET, which was not observed in END. Incorporating strength training into an endurance program can improve time to exhaustion and time trial performance [22, 67, 68, 71, 72]. In agreement, the increase in MAP from WK6 to POST with CET, but not END, highlights the benefit of incorporating resistance exercises to an endurance program for enhancing aerobic performance.

Given the disparities between training regimens and juxtaposition of between-mode recovery amongst studies, it is difficult to attribute the underlying cause of blunted hypertrophy previously observed with concurrent training [18, 20, 23, 24, 73]. One variable that may partially explain diminished hypertrophy with concurrent training is post-exercise protein feeding. Skeletal muscle hypertrophy occurs as a result of repeated and cumulative increases in rates of muscle protein synthesis (MPS) after exercise and ingestion of dietary proteins [34, 45, 74]. We have previously shown protein ingestion following a single bout of concurrent exercise increases acute rates of MPS, while simultaneously attenuating markers of muscle catabolism, compared to a placebo control [34]. Given the importance for dietary protein to enhance muscle growth and remodelling processes, insufficient protein intake around concurrent training sessions may not have maximally stimulated MPS, resulting in the attenuated muscle hypertrophy observed previously [18, 20, 23, 24, 73]. While such a hypothesis is attractive, we acknowledge that without a placebo comparison, we can only speculate on the degree to which protein supplementation facilitated lean mass increases observed in the current investigation. Furthermore, cycling performed in isolation has been shown to induce leg muscle hypertrophy [75], so to what extent protein supplementation influenced the similar post-intervention increase in leg lean mass observed in END compared to CET and RES is unclear.

In contrast to muscle strength and hypertrophy responses, improvements to aspects of muscle power, determined by relative Wingate peak power output, showed a tendency to decrease with CET compared to RES. Previous studies report maximal power output may be more susceptible to impaired development with concurrent training [18, 21, 23, 29, 76]. Compromised power output after concurrent training may be due to impaired rate of force development [21–23, 73, 77] or changes to fiber type [78] and shortening velocity [79]. Force development relies on neural components (e.g. axonal conduction velocity) and myofibre size [80, 81], as well as structural properties (e.g. dystrophin) to transfer force across joints [82]. During muscular contraction, force is transferred from the muscle to tendon both longitudinally [83] as a result of sarcomere shortening and laterally [84] via the extracellular matrix (ECM). Resistance training increases collagen synthesis in the ECM and tendon [85], which, over time, increases tendon cross-sectional area [86, 87] and stiffness [88]. Increases in tendon stiffness are associated with greater torque production and athletic performance [89]. Notably, such adaptations to connective tissue appear to be impaired with concurrent training [87], and may be a source of diminished capacity to generate force rapidly. Similarly, concurrent training can alter fiber type distribution [78], which may result in changes to power development, as optimal shortening velocity and stretch-dependent force differs between fiber types [79]. Given the similar architectural changes between CET and RES in our study, it is possible that CET impaired resistance training-induced adaptations to connective tissue and fiber type distribution, resulting in compromised power outputs.

In contrast to changes in relative Wingate peak power, other measures of power such as the CMJ and SJ were not impaired with CET. This anomaly may be explained by differences in neuromuscular activation between tests. Unlike the single CMJ or SJ, the 30-s all-out Wingate requires coordination of repetitive high-force contractions of antagonistic muscles of the contralateral leg [90]. Given the greater frequency and total volume of exercise, it is possible that Wingate performance may have been attenuated with CET as a function of accumulated fatigue and compromised neuromuscular coordination of repeated high-force contractions. As power-producing capacity is a hallmark of athletic performance [89], future studies incorporating electromyography on multiple muscle groups are needed to monitor fatigue and alterations to neural drive with concurrent training. It should also be noted that the current study may be underpowered to detect appreciable changes in power output as power calculations were based on lean mass change as the primary outcome measure.

Several limitations in the present study are acknowledged. First, without a placebo comparison, limited inferences can be made on whether similar increases in lean mass and strength were due to protein supplementation per se or other factors (i.e. between-session recovery, resistance training program, etc.). Future studies combining concurrent training with protein or placebo supplementation are needed to determine the capacity of protein to directly combat interferences to lean mass and strength. Second, we did not compare alternate modes of endurance training (i.e. cycling vs. running). Given the need for sport specific conditioning, future investigations comparing the incorporation of cycling or running in a concurrent training program are needed to identify if both modalities can be equally compatible with strength training. Finally, we acknowledge that concurrent training bouts cannot always be performed on alternating days; particularly with team sports which often train twice per day [91]. Future studies comparing shorter recovery (i.e. 6–8 h) between sessions in trained athletes are therefore required to optimize adaptations to the demands of same day concurrent training. Similarly, the higher training load associated with concurrent training may increase risk of overtraining and have detrimental impacts on performance outcomes and rates of injury [92]. It is presently unclear whether matching the weekly hours of training between concurrent and single-mode training (i.e. 3 h·wk^−1^) can produce similar degrees of adaptation.

## Conclusion

In conclusion, this is the first investigation to determine the effects of chronic concurrent training in combination with a high protein diet on adaptations to muscle strength, aerobic capacity and maximal power output, as well as lean mass and architectural changes in skeletal muscle. We report that concurrent resistance and endurance training, each performed 3 d·wk^−1^, in the face of a high protein diet, did not impair gains in maximal strength, CMJ, SJ, VO_2peak_, lean mass or muscle architectural changes compared to resistance training alone. However, concurrent training does attenuate improvements to select aspects of lower-body maximal anaerobic power output compared to resistance training, demonstrating a susceptibility in adaptation responses in this paradigm despite recommended optimal protein intake strategies.

### Practical Applications

Our findings provide support for theoretical recommendations for practitioners prescribing concurrent training strategies capable of maximizing strength, hypertrophy and aerobic adaptation responses. First, perform resistance training and endurance training on alternate days to provide sufficient recovery/rest between modes of exercise such that residual fatigue does not limit session intensity [38]. Second, ensure an adequate intake and even distribution of high quality proteins throughout the day, with particular emphasis on intake around exercise [46]. Third, limiting endurance training (where possible) to ~ 30 min per session performed 3 d·wk^−1^ is sufficient to improve aerobic performance without compromising maximal dynamic strength [29].

## Electronic supplementary material

Below is the link to the electronic supplementary material.

Supplementary material 1 (DOCX 43 kb)

Supplementary material 2 (XLSX 15 kb)

Supplementary material 3 (XLSX 13 kb)

Supplementary material 4 (DOCX 25 kb)

Supplementary material 5 (DOCX 199 kb)

Supplementary material 6 (DOCX 65 kb)

Supplementary material 7 (DOCX 146 kb)

Supplementary material 8 (XLSX 338 kb)
