# Supplementary material for: Adaptations to Concurrent Training in Combination with High Protein Availability: A Comparative Trial in Healthy, Recreationally Active Men
Source: Sports Med. 2018 Oct 19;48(12):2869–83. doi: 10.1007/s40279-018-0999-9 (PMC6244626; doi:10.1007/s40279-018-0999-9)
Supplement: Supplementary file 1 — Supplementary material 1 (DOCX 43 kb) [file 40279_2018_999_MOESM1_ESM.docx]

**Online Resource 1**

**Title**: Adaptations to Concurrent Training in Combination with High Protein Availability

**Journal**: Sports Medicine

**Authors**: Baubak Shamim^1^, Brooke L. Devlin^1^, Ryan G. Timmins^2^, Paul J. Tofari^2^, Connor Lee Dow^2^, Vernon G. Coffey^3^, John A Hawley^1^, Donny M. Camera^1^

^1^Exercise and Nutrition Research Program, Mary MacKillop Institute for Health Research, Australian Catholic University, Melbourne, VIC, Australia; ^2^School of Exercise Science, Australian Catholic University, Melbourne, VIC, Australia; ^3^Bond Institute of Health and Sport and Faculty of Health Sciences and Medicine, Bond University, Robina, Queensland, Australia;

**Corresponding author**: Donny Camera, Ph.D.

**Email**: donny.camera@acu.edu.au

**Supplemental Methods**

**Power Testing**

The following performance tests were conducted prior to, and upon completion of the 12-week intervention to determine maximal power output:

*Anaerobic Power Test*

Participants completed a Wingate test ~20 min after VO_2peak_ testing. The Wingate test was performed on an externally loaded stationary cycle ergometer (Monark 894E, Monark, Sweden), with the resistance of the flywheel equivalent to 0.075 kg•kg^-1^ body weight. Participants were familiarized with the test and instructed to remain seated in the saddle for the duration of the test. Briefly, participants began pedaling as fast as possible against the inertial resistance of the ergometer. Once pedaling reached 150 rev•m^-1^, the external load was automatically applied to the flywheel by the ergometer-computer interface (ATS, Monark, Sweden). Participants were verbally encouraged to continue pedaling as hard, and fast as possible throughout the whole 30-s test. Where stringent confines of the test were not met due to participants either rising from saddle (*n* = 1) or incorrect seat position (*n =* 1), data were excluded from analysis.

*Countermovement and Squat Jumps*

Participants completed the countermovement jump (CMJ) and squat jump (SJ) on the force plate prior to IMTP and 1RM testing. Following a standardized warm-up consisting of 5 min of low-intensity cycling (~50 W) and dynamic stretching of the lower limbs, three attempts of each jump were performed, as previously described [1]. For the CMJ, participants started from a standing position and were instructed to maintain their hands on hips throughout the jump. Upon verbal command, participants descended quickly to a self-selected depth then accelerated as rapidly as possible from the bottom position to achieve maximal jump height and velocity. Following the CMJ, SJ’s were performed on the force plate to determine concentric-only jump performance. Participants were instructed to maintain their hands on their hips, squat down to a self-selected depth and hold the position for an audible 3-s count prior to a maximal jump. All efforts were separated by at least 60 s of passive recovery. Variables including jump height (cm), absolute and relative peak power (W and W•kg^-1^) and peak velocity (m•s^-1^) were recorded from the trial with the greatest jump height.

**Architectural Assessment of Vastus Lateralis**

Segmental muscle thickness, pennation angle, fascicle length and volume changes of the VL were assessed from ultrasound images taken along the longitudinal axis of the muscle belly utilizing a two dimensional, B-mode ultrasound (frequency, 12 Mhz; depth, 8 cm; field of view, 14 x 47 mm; GE Healthcare Vivid-i, USA) at baseline, after weeks 2, 4, 8, and post-intervention. Images were taken at 75 (proximal), 50 (mid), and 33% (distal) of the distance between the central palpable point of the greater trochanter and the lateral condyle of the femur. Once the scanning sites were determined, the distances from various anatomical landmarks were recorded to ensure reproducibility for future testing sessions. These landmarks included the ischial tuberosity, fibula head and the greater trochanter. On subsequent visits the scanning sites were determined and marked on the skin and then confirmed by replicated landmark distance measures. All architectural assessments were performed with participants in a supine position with the hip and knee in a neutral position following at least 5 min of inactivity and prior to any testing or exercise sessions. To obtain ultrasound images, the linear array ultrasound probe was aligned parallel to the muscle fascicles and perpendicular to the skin with a layer of conductive gel. Care was taken to ensure minimal pressure was placed on the skin by the probe as this may influence measurement accuracy [2]. Finally, the probe orientation was manipulated slightly by the assessor if the superficial and deep aponeuroses were not parallel. Two images were taken at each site, and the best quality image used for subsequent analysis.

Once the images were collected, analysis was undertaken off-line (MicroDicom version 0.7.8, Bulgaria). For each image and site, fascicle length estimation was performed as described elsewhere [3,4]. At each site, muscle thickness was defined as the distance between the superficial and deep aponeuroses of the VL. A fascicle of interest was outlined and marked on the image, and the angle at which it inserted onto the deep aponeurosis was determined as the pennation angle. The superficial and deep aponeurosis angles were determined as the angle between the line marked as the aponeurosis and an intersecting horizontal reference line across the captured image [3,4].

The same assessor collected and analysed all scans and was blinded to participant identifiers (name and group) during the collection and analysis of the images. Day-to-day reliability of the assessor was determined prior to data collection in a small pilot study (*n* = 9). Measures of reliability included intraclass correlation coefficients (ICC), typical error (TE) and TE as a coefficient of variation (%TE). Minimum detectable change at a 95% confidence interval (MDC_95_) was calculated as TE x 1.96 x √2. Based on previous quantitative reliability literature, it was determined that an ICC ≥ 0.90 was regarded as high, between 0.80 and 0.89 as moderate and ≤0.79 as poor. A %TE ≤10% was considered to represent an acceptable level of reliability. Assessments for the pilot study were taken one day apart at the same time of day. Across all three sites for muscle thickness ICCs ranged from 0.97 to 0.99, TE from 0.09 to 0.22, %TE from 1.0 to 3.9% and MDC_95_ from 0.25 to 0.61cm. For pennation angle ICCs ranged from 0.90 to 0.98, TE from 0.16 to 0.33, %TE from 2.1 to 4.0% and MDC_95_ from 0.44° to 0.91°. For fascicle length ICCs ranged from 0.90 to 0.98, TE from 0.18 to 0.30, %TE from 3.9 to 4.9% and MDC_95_ from 0.49 cm to 0.83 cm.

Muscle thickness measures from ultrasound have been used to estimate muscle volume at a single time point and following training interventions [5–7]. In the current study, thigh length and VL thickness measures at the mid-point of the thigh were utilised with the following validated equation [5] to estimate muscle volume:

MV (cm^3^) = (MT x 311.732) + (TL x 53.346) – 2058.529

Where MV = muscle volume, MT = muscle thickness in centimeters, and TL = thigh length in centimeters.

**Diet**

A free-living, high-protein (2 g•kg^-1^•d^-1^) eating plan was implemented over the 12-wk intervention. Energy intake was based on the Cunningham Equation (using fat free mass from DXA) and Physical Activity Level (PAL) of 1.6 (RES and END) or 1.8 (CET), and modified accordingly depending on individual weight changes over the 12-wk intervention. Macronutrient composition was monitored throughout the intervention with total energy intake (TEI) and protein intake a focus. Carbohydrate and fat intake were recommended to be within the Acceptable Macronutrient Distribution Range for these macronutrients (45-65% and 20-35% TEI for carbohydrate and fat, respectively). Participants were instructed to remain in a positive energy balance to mitigate any potential of energetic stress related interferences to anabolic adaptations [8,9].

Prior to and throughout the intervention, participants were provided the following guidelines to reach protein and energy targets: 1) distribute protein intake evenly throughout the day across 4-6 meals [10,11] and 2) consume ~20-30 g of protein prior to bed to maximize potential for muscle protein synthesis [12–14]. Participants were provided with ~34 g of whey protein (Pure Warrior 100% WPI, Swisse™, Australia) following every training session to maximally stimulate post-exercise rates of muscle protein synthesis [15]. In addition, all participants were provided with a whey protein supplement (Whey Protein Concentrate, Bulk Nutrients, Australia) to consume as needed throughout the 12-wk intervention. Both protein supplements are commercially available and undergo batch testing for banned substances by independent organizations in compliance with the World Anti-Doping Authority. To further assist in reaching the 2 g•kg^-1^•d^-1^ protein amount, participants were also provided with weekly allotments of yoghurt from Chobani (Chobani LLC, Australia) and Jalna (Jalna Dairy Foods Pty Ltd, Australia), as well as almonds (Almond Board of Australia).

Participants attended consultations with an Accredited Practicing Dietitian on a fortnightly basis for a total of 8 consultations (Baseline prior to intervention, week 1, 3, 5, 7, 9, and 11, as well as week 13 to conclude the study). Consultations lasted ~20-30 min and provided participants with education, support, and advice to ensure nutrient targets were met, protein intake was evenly distributed throughout the day, and to monitor, and assess, dietary adherence. Advice was tailored and individualized to each participant depending on food preference, as well as eating habits and behaviours. On alternate weeks, the Accredited Practicing Dietitian contacted participants via text message and phone call to ensure dietary compliance and food record maintenance. Daily food records were kept by participants through mobile phone applications Easy Diet Diary (Xyris Software Pty Ltd, Australia) for participants with iPhones^®^ (Apple Inc., USA; *n* = 20) and MyFitnessPal (MyFitnessPal Inc., USA) for participants with Android-based (Google Inc., USA) devices (*n* = 12). All dietary intake data was analyzed using FoodWorks 8^©^ (Xyris Software Pty Ltd, Australia) to ensure the same food database was used for all analysis. Diet records were analyzed for energy (kJ•kg^-1^), protein, carbohydrate, and fat (g•kg^-1^ for all macronutrients) to provide a daily average for the entire 12-week intervention. Habitual dietary intake at baseline was assessed prior to commencing the study and analyzed for energy and macronutrient intakes.

**References**

1. Tofari P, Kemp J, Cormack S. A Self-Paced Team Sport Match Simulation Results In Reductions In Voluntary Activation And Modifications To Biological, Perceptual And Performance Measures At Half-Time, And For Up To 96 Hours Post-Match. J Strength Cond Res. 2017;

2. Klimstra M, Dowling J, Durkin JL, MacDonald M. The effect of ultrasound probe orientation on muscle architecture measurement. J Electromyogr Kinesiol. 2007;17:504–14.

3. Blazevich AJ, Gill ND, Zhou S. Intra- and intermuscular variation in human quadriceps femoris architecture assessed in vivo. J Anat. 2006;209:289–310.

4. Kellis E, Galanis N, Natsis K, Kapetanos G. Validity of architectural properties of the hamstring muscles: correlation of ultrasound findings with cadaveric dissection. J Biomech. 2009;42:2549–54.

5. Miyatani M, Kanehisa H, Kuno S, Nishijima T, Fukunaga T. Validity of ultrasonograph muscle thickness measurements for estimating muscle volume of knee extensors in humans. Eur J Appl Physiol. 2002;86:203–8.

6. Miyatani M, Kanehisa H, Ito M, Kawakami Y, Fukunaga T. The accuracy of volume estimates using ultrasound muscle thickness measurements in different muscle groups. Eur J Appl Physiol. 2004;91:264–72.

7. Franchi MV, Longo S, Mallinson J, Quinlan JI, Taylor T, Greenhaff PL, et al. Muscle thickness correlates to muscle cross-sectional area in the assessment of strength training-induced hypertrophy. Scand J Med Sci Sports. 2017;

8. Perez-Schindler J, Hamilton DL, Moore DR, Baar K, Philp A. Nutritional strategies to support concurrent training. Eur J Sport Sci. 2015;15:41–52.

9. Hamilton DL, Philp A. Can AMPK mediated suppression of mTORC1 explain the concurrent training effect? Cell Mol Exerc Physiol. 2013;2:e4.

10. Areta JL, Burke LM, Ross ML, Camera DM, West DWD, Broad EM, et al. Timing and distribution of protein ingestion during prolonged recovery from resistance exercise alters myofibrillar protein synthesis. J Physiol. 2013;591:2319–31.

11. Loenneke JP, Loprinzi PD, Murphy CH, Phillips SM. Per meal dose and frequency of protein consumption is associated with lean mass and muscle performance. Clin Nutr Edinb Scotl. 2016;35:1506–11.

12. Moore DR, Robinson MJ, Fry JL, Tang JE, Glover EI, Wilkinson SB, et al. Ingested protein dose response of muscle and albumin protein synthesis after resistance exercise in young men. Am J Clin Nutr. 2009;89:161–8.

13. Snijders T, Res PT, Smeets JS, van Vliet S, van Kranenburg J, Maase K, et al. Protein Ingestion before Sleep Increases Muscle Mass and Strength Gains during Prolonged Resistance-Type Exercise Training in Healthy Young Men. J Nutr. 2015;145:1178–84.

14. Trommelen J, Kouw IWK, Holwerda AM, Snijders T, Halson SL, Rollo I, et al. Pre-sleep dietary protein-derived amino acids are incorporated in myofibrillar protein during post-exercise overnight recovery. Am J Physiol - Endocrinol Metab. 2017;ajpendo.00273.2016.

15. Macnaughton LS, Wardle SL, Witard OC, McGlory C, Hamilton DL, Jeromson S, et al. The response of muscle protein synthesis following whole‐body resistance exercise is greater following 40 g than 20 g of ingested whey protein. Physiol Rep. 2016;4:e12893.
