# Supplementary material for: Adaptations to Concurrent Training in Combination with High Protein Availability: A Comparative Trial in Healthy, Recreationally Active Men
Source: Sports Med. 2018 Oct 19;48(12):2869–83. doi: 10.1007/s40279-018-0999-9 (PMC6244626; doi:10.1007/s40279-018-0999-9)
Supplement: Supplementary file 4 — Supplementary material 4 (DOCX 25 kb) [file 40279_2018_999_MOESM4_ESM.docx]

**Online Resource 4: Changes to vastus lateralis muscle architecture throughout the 12 wk training intervention measured by two-dimensional B-mode ultrasound.** Values are presented as means ± SD. a = *P* < 0.05 from PRE. b = *P* < 0.05 from WK2. c = *P* < 0.05 from WK4. d = *P* < 0.05 from WK8. ǂ = *P* < 0.05 from END at time point. (ǂ) = *P* < 0.055 from END at time point. Abbreviations: CET, concurrent exercise training; RES, resistance training; END, endurance training.

|  |  |  |  |  |  |  |  |  |  |  |  |  |  |  |  |
| --- | --- | --- | --- | --- | --- | --- | --- | --- | --- | --- | --- | --- | --- | --- | --- |
|  |  |  | **Measure** | | | | | | | | | | | | |
|  | |  | **Thickness (cm)** | | |  | **Pennation (°)** | | |  | **Fascicle (cm)** | | |  | **Estimated Volume (cm^3^)** |
| *Group* | *Proximal* | | | *Middle* | *Distal* |  | *Proximal* | *Middle* | *Distal* |  | *Proximal* | *Middle* | *Distal* |  |  |
| *PRE* | **CET** | | 2.8 ± 0.4 | 2.7 ± 0.4 | 2.0 ± 0.4 |  | 18.5 ± 2.4 | 18.3 ± 1.6 | 17.4 ± 2.5 |  | 9.3 ± 0.8 | 9.0 ± 0.9^ǂ^ | 7.1 ± 0.9 |  | 956 ± 164 |
|  | **RES** | | 2.7 ± 0.3 | 2.6 ± 0.2 | 2.0 ± 0.3 |  | 18.2 ± 2.1 | 18.0 ± 1.3 | 16.2 ± 0.7 |  | 8.9 ± 0.6 | 8.9 ± 0.8 | 7.4 ± 0.9 |  | 990 ± 122 |
|  | **END** | | 2.6 ± 0.3 | 2.4 ± 0.2 | 1.9 ± 0.2 |  | 17.3 ± 1.3 | 17.5 ± 2.1 | 16.3 ± 1.7 |  | 9.2 ± 1.0 | 8.3 ± 0.9 | 7.2 ± 0.6 |  | 940 ± 124 |
|  |  |  |  |  |  |  |  |  |  |  |  |  |  |  |  |
| *WK2* | **CET** | | 3.0 ± 0.4^aǂ^ | 3.0 ± 0.4^aǂ^ | 2.4 ± 0.4^aǂ^ |  | 20.1 ± 2.1^a^ | 19.8 ± 2.3^aǂ^ | 18.6 ± 2.4^a^ |  | 9.1 ± 0.6 | 9.1 ± 0.6 | 8.0 ± 0.6^a^ |  | 1040 ± 163^a^ |
|  | **RES** | | 2.9 ± 0.3^a^ | 2.8 ± 0.3^a^ | 2.2 ± 0.3^a^ |  | 19.2 ± 2.1 | 18.8 ± 2.3 | 18.1 ± 2.4^a^ |  | 9.1 ± 0.4 | 9.0 ± 0.6 | 7.6 ± 0.7 |  | 1040 ± 133^a^ |
|  | **END** | | 2.7 ± 0.2 | 2.5 ± 0.3 | 2.1 ± 0.1^a^ |  | 18.4 ± 1.5 | 17.6 ± 1.4 | 16.9 ± 1.1 |  | 8.8 ± 0.6 | 8.6 ± 0.6 | 7.4 ± 0.6 |  | 966 ± 133 |
|  |  |  |  |  |  |  |  |  |  |  |  |  |  |  |  |
| *WK4* | **CET** | | 3.1 ± 0.4^aǂ^ | 3.0 ± 0.6^aǂ^ | 2.4 ± 0.4^a^ |  | 20.3 ± 2.3^a^ | 19.9 ± 2.2^a^ | 19.1 ± 3.2^a^ |  | 9.2 ± 0.9^ǂ^ | 9.2 ± 0.7 | 7.6 ± 0.7^a^ |  | 1060 ± 171^a^ |
|  | **RES** | | 2.9 ± 0.3^aǂ^ | 2.8 ± 0.3^a^ | 2.2 ± 0.3^a^ |  | 19.5 ± 2.1^a^ | 19.4 ± 2.1^a^ | 17.7 ± 2.6^a^ |  | 9.1 ± 0.5^(ǂ)^ | 8.9 ± 0.6 | 7.8 ± 0.9 |  | 1060 ± 129^a^ |
|  | **END** | | 2.6 ± 0.2 | 2.6 ± 0.2^a^ | 2.1 ± 0.2^a^ |  | 18.6 ± 1.6 | 18.0 ± 1.1 | 17.3 ± 1.9 |  | 8.5 ± 0.6^a^ | 8.6 ± 0.7 | 7.3 ± 0.7 |  | 992 ± 115^a^ |
|  |  |  |  |  |  |  |  |  |  |  |  |  |  |  |  |
| *WK8* | **CET** | | 3.2 ± 0.4^abǂ^ | 3.1 ± 0.4^abǂ^ | 2.5 ± 0.5^aǂ^ |  | 21.6 ± 2.1^abc^ | 21.0 ± 2.6^a^ | 20.1 ± 3.5^ab^ |  | 9.2 ± 0.7^ǂ^ | 9.2 ± 0.6^ǂ^ | 7.7 ± 1.0^a^ |  | 1090 ± 166^abc^ |
|  | **RES** | | 3.0 ± 0.3^abcǂ^ | 2.9 ± 0.3^abǂ^ | 2.3 ± 0.4^a^ |  | 20.9 ± 1.8^abc^ | 20.5 ± 1.8^ab^ | 18.4 ± 2.4^a^ |  | 8.9 ± 0.7^ǂ^ | 8.7 ± 0.6 | 7.6 ± 1.2 |  | 1080 ± 132^ab^ |
|  | **END** | | 2.7 ± 0.2^a^ | 2.6 ± 0.3^ab^ | 2.1 ± 0.2^a^ |  | 20.0 ±2.1^abc^ | 19.1 ± 2.1^abc^ | 18.0 ± 1.5^a^ |  | 8.3 ± 0.8^a^ | 8.4 ± 0.7 | 7.2 ± 0.9 |  | 1010 ± 135^ab^ |
|  |  |  |  |  |  |  |  |  |  |  |  |  |  |  |  |
| *POST* | **CET** | | 3.2 ± 0.4^abcǂ^ | 3.2 ± 0.4^abcǂ^ | 2.4 ± 0.4 |  | 21.5 ± 2.3^abc^ | 20.5 ± 1.9^a^ | 19.5 ± 3.2^a^ |  | 9.3 ± 0.7^ǂ^ | 9.5 ± 0.5^ǂ^ | 7.8 ± 0.8^a^ |  | 1100 ± 162^abc^ |
|  | **RES** | | 3.1 ± 0.3^abcǂ^ | 3.0 ± 0.3^abcǂ^ | 2.4 ± 0.3 |  | 20.9 ± 1.7^abc^ | 20.1 ± 1.9^ab^ | 19.3 ± 1.6^ac^ |  | 9.0 ± 0.5 | 9.1 ± 0.5^ǂ^ | 7.7 ± 1.1 |  | 1100 ± 134^abc^ |
|  | **END** | | 2.7 ± 0.2^ac^ | 2.6 ± 0.2^ab^ | 2.1 ± 0.2 |  | 19.8 ± 2.3^abc^ | 19.8 ± 2.0^abc^ | 18.3 ± 1.6^a^ |  | 8.5 ± 0.6^a^ | 8.1 ± 0.6 | 7.1 ± 0.7 |  | 1010 ± 115^ab^ |

**Online Resource 4**

**Title**: Adaptations to Concurrent Training in Combination with High Protein Availability

**Journal**: Sports Medicine

**Authors**: Baubak Shamim^1^, Brooke L. Devlin^1^, Ryan G. Timmins^2^, Paul J. Tofari^2^, Connor Lee Dow^2^, Vernon G. Coffey^3^, John A Hawley^1^, Donny M. Camera^1^

^1^Exercise and Nutrition Research Program, Mary MacKillop Institute for Health Research, Australian Catholic University, Melbourne, VIC, Australia; ^2^School of Exercise Science, Australian Catholic University, Melbourne, VIC, Australia; ^3^Bond Institute of Health and Sport and Faculty of Health Sciences and Medicine, Bond University, Robina, Queensland, Australia;

**Corresponding author**: Donny Camera, Ph.D.

**Email**: donny.camera@acu.edu.au
