# Supplementary material for: Adaptations to Concurrent Training in Combination with High Protein Availability: A Comparative Trial in Healthy, Recreationally Active Men
Source: Sports Med. 2018 Oct 19;48(12):2869–83. doi: 10.1007/s40279-018-0999-9 (PMC6244626; doi:10.1007/s40279-018-0999-9)
Supplement: Supplementary file 6 — Supplementary material 6 (DOCX 65 kb) [file 40279_2018_999_MOESM6_ESM.docx]

**Online Resource 6**

**Title**: Adaptations to Concurrent Training in Combination with High Protein Availability

**Journal**: Sports Medicine

**Authors**: Baubak Shamim^1^, Brooke L. Devlin^1^, Ryan G. Timmins^2^, Paul J. Tofari^2^, Connor Lee Dow^2^, Vernon G. Coffey^3^, John A Hawley^1^, Donny M. Camera^1^

^1^Exercise and Nutrition Research Program, Mary MacKillop Institute for Health Research, Australian Catholic University, Melbourne, VIC, Australia; ^2^School of Exercise Science, Australian Catholic University, Melbourne, VIC, Australia; ^3^Bond Institute of Health and Sport and Faculty of Health Sciences and Medicine, Bond University, Robina, Queensland, Australia;

**Corresponding author**: Donny Camera, Ph.D.

**Email**: donny.camera@acu.edu.au

**Online Resource 6: Session average for training variables throughout the 12 wk training intervention.** ǂ = *P* < 0.05 from END. ^ = *P* < 0.05 from RES. Abbreviations: CET, concurrent exercise training; RES, resistance training; END, endurance training.

|  |  |  |  |  |  |
| --- | --- | --- | --- | --- | --- |
|  |  | **Training Group** | | |  |
| **Variable** | | **CET** | **RES** | **END** |  |
| *Resistance Variables* | |  |  |  |  |
|  | Time To Complete Set (s●set^-1^) | 42 ± 9 | 40 ± 3 | - |  |
|  | Rest Interval (s) | 185 ± 2 | 185 ± 3 | - |  |
|  | Rating of Perceived Exertion | 7 ± 0 | 7 ± 1 | - |  |
| *Endurance Variables* | |  |  |  |  |
|  | Training Hours (h●wk^-1^) | 1.44 ± 0.02 | - | 1.43 ± 0.04 |  |
|  | Heart Rate (bpm) | 159 ± 7 | - | 155 ± 8 |  |
|  | Rating of Perceived Exertion | 6 ± 1^ǂ^ | - | 5 ± 1 |  |
|  |  |  |  |  |  |
| Time Between Session (h) | | 23.6 ± 0.85^^ǂ^ | 47.7 ± 3.13 | 48.0 ± 1.76 |  |
|  |  |  |  |  |  |
