# Supplementary material for: Adaptations to Concurrent Training in Combination with High Protein Availability: A Comparative Trial in Healthy, Recreationally Active Men
Source: Sports Med. 2018 Oct 19;48(12):2869–83. doi: 10.1007/s40279-018-0999-9 (PMC6244626; doi:10.1007/s40279-018-0999-9)
Supplement: Supplementary file 7 — Supplementary material 7 (DOCX 146 kb) [file 40279_2018_999_MOESM7_ESM.docx]

**Online Resource 7**

**Title**: Adaptations to Concurrent Training in Combination with High Protein Availability

**Journal**: Sports Medicine

**Authors**: Baubak Shamim^1^, Brooke L. Devlin^1^, Ryan G. Timmins^2^, Paul J. Tofari^2^, Connor Lee Dow^2^, Vernon G. Coffey^3^, John A Hawley^1^, Donny M. Camera^1^

^1^Exercise and Nutrition Research Program, Mary MacKillop Institute for Health Research, Australian Catholic University, Melbourne, VIC, Australia; ^2^School of Exercise Science, Australian Catholic University, Melbourne, VIC, Australia; ^3^Bond Institute of Health and Sport and Faculty of Health Sciences and Medicine, Bond University, Robina, Queensland, Australia;

**Corresponding author**: Donny Camera, Ph.D.

**Email**: donny.camera@acu.edu.au


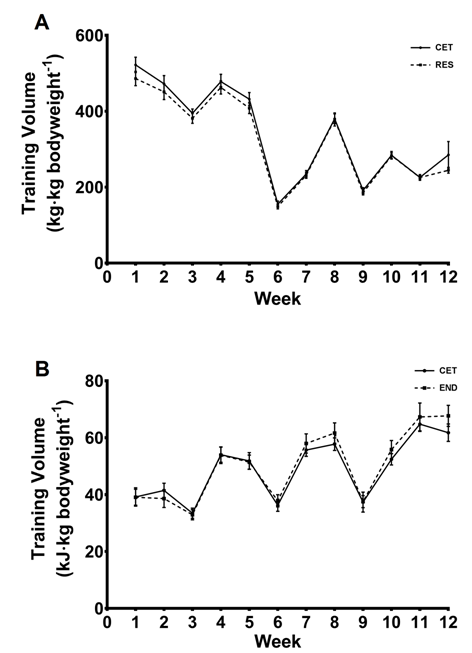


**Online Resource 7: Weekly average training volume for A) resistance-based and B) endurance-based training programs.** Abbreviations: CET, concurrent exercise training; RES, resistance training; END, endurance training.
